# Fine-tuned nitric oxide and hormone interface in plant root development and regeneration

**DOI:** 10.1093/jxb/erac508

**Published:** 2022-12-22

**Authors:** Alvaro Sanchez-Corrionero, Inmaculada Sánchez-Vicente, Noelia Arteaga, Isabel Manrique-Gil, Sara Gómez-Jiménez, Isabel Torres-Quezada, Pablo Albertos, Oscar Lorenzo

**Affiliations:** Departamento de Botánica y Fisiología Vegetal, Instituto de Investigación en Agrobiotecnología (CIALE), Facultad de Biología, Universidad de Salamanca, C/ Río Duero 12, 37185 Salamanca, Spain; Universidad Politécnica de Madrid, Madrid, Spain; Departamento de Botánica y Fisiología Vegetal, Instituto de Investigación en Agrobiotecnología (CIALE), Facultad de Biología, Universidad de Salamanca, C/ Río Duero 12, 37185 Salamanca, Spain; Departamento de Botánica y Fisiología Vegetal, Instituto de Investigación en Agrobiotecnología (CIALE), Facultad de Biología, Universidad de Salamanca, C/ Río Duero 12, 37185 Salamanca, Spain; Departamento de Botánica y Fisiología Vegetal, Instituto de Investigación en Agrobiotecnología (CIALE), Facultad de Biología, Universidad de Salamanca, C/ Río Duero 12, 37185 Salamanca, Spain; Departamento de Botánica y Fisiología Vegetal, Instituto de Investigación en Agrobiotecnología (CIALE), Facultad de Biología, Universidad de Salamanca, C/ Río Duero 12, 37185 Salamanca, Spain; Departamento de Botánica y Fisiología Vegetal, Instituto de Investigación en Agrobiotecnología (CIALE), Facultad de Biología, Universidad de Salamanca, C/ Río Duero 12, 37185 Salamanca, Spain; Departamento de Botánica y Fisiología Vegetal, Instituto de Investigación en Agrobiotecnología (CIALE), Facultad de Biología, Universidad de Salamanca, C/ Río Duero 12, 37185 Salamanca, Spain; Departamento de Botánica y Fisiología Vegetal, Instituto de Investigación en Agrobiotecnología (CIALE), Facultad de Biología, Universidad de Salamanca, C/ Río Duero 12, 37185 Salamanca, Spain; Universidad de Sevilla, Spain

**Keywords:** Gasotransmitter, hypoxia, phytoglobins, phytohormones, root apical meristem, stem cell niche

## Abstract

Plant root growth and developmental capacities reside in a few stem cells of the root apical meristem (RAM). Maintenance of these stem cells requires regenerative divisions of the initial stem cell niche (SCN) cells, self-maintenance, and proliferative divisions of the daughter cells. This ensures sufficient cell diversity to guarantee the development of complex root tissues in the plant. Damage in the root during growth involves the formation of a new post-embryonic root, a process known as regeneration. Post-embryonic root development and organogenesis processes include primary root development and SCN maintenance, plant regeneration, and the development of adventitious and lateral roots. These developmental processes require a fine-tuned balance between cell proliferation and maintenance. An important regulator during root development and regeneration is the gasotransmitter nitric oxide (NO). In this review we have sought to compile how NO regulates cell rate proliferation, cell differentiation, and quiescence of SCNs, usually through interaction with phytohormones, or other molecular mechanisms involved in cellular redox homeostasis. NO exerts a role on molecular components of the auxin and cytokinin signaling pathways in primary roots that affects cell proliferation and maintenance of the RAM. During root regeneration, a peak of auxin and cytokinin triggers specific molecular programs. Moreover, NO participates in adventitious root formation through its interaction with players of the brassinosteroid and cytokinin signaling cascade. Lately, NO has been implicated in root regeneration under hypoxia conditions by regulating stem cell specification through phytoglobins.

## Teaser

This review provides physiological and molecular insights into the control of root development and regeneration by the gasotransmitter nitric oxide (NO) with special emphasis on the interaction with phytohormones and hypoxia-related stress.

## Introduction

### Post-embryonic root developmental processes

The architecture of the root system dramatically influences agricultural production, and interest in this organ in breeding programs has increased through root functional traits to develop crops with higher nutrient and water uptake efficiency (Lynch, 2019). *Arabidopsis thaliana* root is composed of three major zones comprising the meristematic zone (MZ), elongation zone (EZ), and differentiation zone (DZ). The MZ includes the root apical meristem (RAM) composed of stem cells surrounding a small group of cells with low mitotic activity, and the quiescent center (QC), which gives rise to the different root tissues, ensuring continuous root growth (Aichinger *et al.*, 2012; Petricka *et al.*, 2012). This capacity requires regenerative divisions of the initial cells of the stem cell niche (SCN), self-maintenance, and proliferative divisions of the daughter cells that result in the different cell types of the root to provide sufficient cell diversity and guarantee the development of complex tissues (Gutierrez, 2005).

However, during post-embryonic development, several types of roots can be generated (Motte *et al.*, 2019), such as lateral roots (LRs) or adventitious roots (ARs). LRs are constantly formed and appear at different developmental stages along the main root. The organogenesis of LRs has been classified into four steps (Du and Scheres, 2018). First, LR positioning, which occurs mainly in the oscillation zone (OZ), integrates signals for positioning, specification, and activation of lateral root founder cells (LRFCs), and regulates the distribution of LRs. A second step includes LR initiation through nuclear migration of LRFCs until the first asymmetric division. Third, LR development in the DZ is controlled by intrinsic signals. Fourth, LR emergence occurs in the DZ, as an interactive process between the LR primordia and the surrounding tissues. Once the LR has emerged, the cellular and tissular distribution is very similar to that of the main root (Xuan *et al.*, 2020).

Under normal growth conditions, self-regeneration (maintenance) ensures the replacement of stem cells during post-embryonic organogenesis, allowing for proper development. However, when the SCN is damaged by biotic or abiotic agents, the stem cells are rapidly replaced or regenerated (Efroni *et al.*, 2016). Organ regeneration after an injury in plants or animals requires the activation of different developmental programs such as cell dedifferentiation or cell proliferation until a new organ is formed (King and Newmark, 2012). Regeneration is the ability of multicellular organisms to reconstitute or develop new cells, tissues, or whole organs after damage or injury (Birnbaum and Sánchez-Alvarado, 2008). In plants, regeneration is particularly important as it guarantees adaptation to a changing environment (Lup *et al.*, 2016). Regeneration can occur from numerous plant tissues and is controlled at the hormonal and genetic levels. Auxin (Aux) and cytokinin (CK) play a dominant role in regeneration, and their balance is decisive for the specification of new meristems (Sugimoto *et al.*, 2011).

### Bases for nitric oxide action during root development

Nitric oxide (NO) is an essential gasotransmitter for the regulation of development and the response to various stresses (Albertos *et al.*, 2016; Sánchez-Vicente *et al.*, 2019). Due to its chemical nature, NO can react with different molecules and modify the signaling pathway of different phytohormones, reshaping plant responses. NO has a highly dynamic spatio-temporal pattern and is rapidly synthesized and scavenged to modify specific targets that tailor the redox balance of the cell.

NO homeostasis is of main relevance for controlling physiological events during the life cycle of plants, since effects are often associated with the levels of this signaling molecule. In plants, the mechanisms described to modulate NO levels are complex and diverse. To date, different possible synthesis pathways have been described, which can be classified as reductive and oxidative (Gupta and Igamberdiev, 2011). In roots, the reductive pathway appears to be necessary to NO production and gathers together the role of NI-NOR (nitrite-NO oxidoreductase), the plasma membrane-bound nitrate reductase (PM-NR) (Stöhr *et al.*, 2001; Stöhr and Stremlau, 2006), mitochondrial electron transport (Planchet *et al.*, 2005), and the nitrate reductase (NR) (Vázquez *et al.*, 2019). Remarkably, the functioning of this pathway is mandatory during periods of soil flooding. In this environmental context, nitrate is reduced to nitrite, and reductive enzyme reactions are very active, promoting a burst of NO (Planchet *et al.*, 2005; Hebelstrup *et al.*, 2012), which is balanced through the action of phytoglobins (PGBs) (Hebelstrup *et al.*, 2012; Hartman *et al.*, 2019). In addition, NIN-like protein7 (NLP7) regulates nitrate assimilation and, together with PROTEOLYSIS6 (PRT6), participates in a regulatory loop that controls NO homeostasis and action (Castillo *et al.*, 2021). Another component involved in NO control is related to prohibitins (PHBs). The function of the PHB family is unclear, but, as in animals, plant PHBs are targeted to the mitochondria, where they are implicated in their biogenesis and function (Ahn *et al.*, 2006; Van Aken *et al.*, 2007). Specifically, the mitochondrial protein PHB3 is described as a component involved in NO production and NO-mediated effects in roots under NaCl stress (Wang *et al.*, 2010).

The maintenance of NO levels is essential for the proper regulation of the cellular redox state, and two main mechanisms have been described that contribute to NO homeostasis. The first is related to the formation of *S*-nitrosothiols (SNOs) triggered by the reaction of NO with reduced thiols and regulated by the action of the enzyme *S*-nitrosoglutathione reductase (GSNOR) (Sakamoto *et al.*, 2002; Leterrier *et al.*, 2011). In addition, the involvement of the aldo-keto reductase (AKR) superfamily of proteins has recently been described as an NADPH-dependent component linked to mammalian NO and GSNO metabolism (Treffon *et al.*, 2021). The second is the function of PGBs as NO-detoxifying/scavenging proteins, essential during oxygen depletion responses to reset the cellular redox state, as mentioned above (Hebelstrup *et al.*, 2012; Hartman *et al.*, 2019).

NO exerts its action mainly through post-translational modifications (PTMs), with *S*-nitrosylation of cysteine residues being the most relevant (Hess *et al.*, 2001; Astier and Lindermayr, 2012; Kovacs and Lindermayr, 2013). This PTM can promote conformational changes in the protein structure, affecting its stability, activity, and/or localization (Hess *et al.*, 2005; Albertos *et al.*, 2015; Feng *et al.*, 2019).

At a physiological level, NO can orchestrate internal and external signals to adjust the growth, patterning, and differentiation of the root architecture. These include the three-dimensional configuration in the primary root (PR) and the post-embryonic developed branching pattern composed of the LR and AR. To fine-tune root organogenesis, NO regulates specific targets, coordinating a network composed of the main hormonal pathways involved in the control of the root system, including Aux, brassinosteroids (BRs), CKs, and strigolactones (SLs).

In *A. thaliana*, cell proliferation and differentiation are safely regulated by several intrinsic and extrinsic signaling pathways, most notably Aux/CKs and reactive oxygen species (ROS) homeostasis (Wany *et al.*, 2018). Among reactive nitrogen species (RNS), NO has been identified as an important regulator of plant growth and development (Sanz *et al.*, 2015), promoting cell differentiation and reducing cell divisions in the RAM (Fernández-Marcos *et al.*, 2011). Thus, this review brings together current knowledge on the functions of NO during PR development, AR and LR formation, SCN maintenance and regeneration, and its interaction with the plant hormones Aux, CKs, BRs, or SLs, including a final note on the emerging impact on these processes in hypoxia-related stress.

## Overall involvement of nitric oxide in roots

### Primary root development and stem cell niche maintenance

**Fig. 1.**
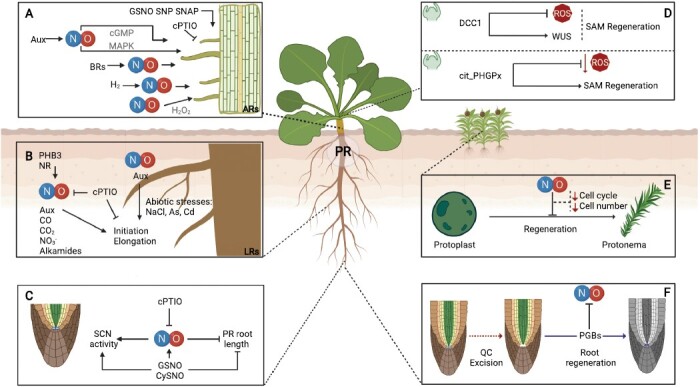
NO regulation of post-embryonic development. (A) Promotion of AR development by exogenous NO through pharmacological donors (GSNO, SNP, and SNAP) and reduction by the NO scavenger cPTIO in cucumber, *Panax ginseng*, *Vigna radiata*, and mung bean. NO mediates the Aux response during AR formation through a cGMP-dependent or cGMP-independent pathway, where a MAPK cascade is involved in the latter. Also, NO synthesis is induced by BRs, increasing AR development. In addition, hydrogen gas (H_2_) mediated by NO promotes AR formation in cucumber, and NO induction of ARs is mediated by hydrogen peroxide (H_2_O_2_) in mung bean and chrysanthemum. (B) NO promotes LR initiation and growth, both under normal conditions and in response to abiotic stresses in different species. Other molecules have also been described to induce LR formation in close association with NO, such as Aux, carbon monoxide (CO), alkamides, carbon dioxide (CO_2_), and nitrate (NO_3_). PHB3 and NR have been described as key NO sources during LR development. (C) NO affects PR length negatively while causing an increase of SCN activity. This effect is reversed when NO is scavenged by cPTIO. (D) Reactive oxygen species (ROS) involvement in shoot apical meristem (SAM) regeneration. DDC1 regulates WUS, promoting SAM regeneration through the reduction of ROS levels in *Arabidopsis thaliana*, and phospholipid hydroperoxide glutathione peroxidase (PHGPx) decreases the ROS levels, promoting SAM regeneration in leaf disks of *Nicotiana tabaccum*. (E) NO reduces protonema regeneration from protoplasts in *Physcomitrella patens* through the reduction of cell cycle and protonema cells. (F) *Zea mays de novo* root organogenesis after the surgical removal of the quiescent center (QC) mediated by phytoglobins (PGBs) and regulation of NO (created with BioRender.com).

Several studies have shown that ROS and RNS critically regulate the process of RAM development, mainly through crosstalk between NO and Aux during root formation and growth (Wany *et al.*, 2018; Prakash *et al.*, 2020) (Fig. 1A–F). NO was introduced as a promoter of root elongation, as it was observed that solutions containing substances that release NO non-enzymatically induced root tip expansion in a dose-dependent manner in maize root segments (Gouvêa *et al.*, 1997). There is also evidence of an inducing effect of NO on LRs while decreasing PR length in a dose-dependent manner in tomato (Correa-Aragunde *et al.*, 2004). Furthermore, Auxs have been shown to play a central role in PR growth and development (Xu *et al.*, 2006; Yu *et al.*, 2014; Olatunji *et al.*, 2017; Shahid *et al.*, 2019), revealing indole-3-acetic acid (IAA) as a master regulator of root architecture and development (Saini *et al.*, 2013). Moreover, NO has been reported to regulate Aux-mediated root growth and development (Fernández-Marcos *et al.*, 2012; Yu *et al.*, 2014) and has implications in the division and differentiation of the cells located in the PR meristem (Fernández-Marcos *et al.*, 2011; Sanz *et al.*, 2014).

Different approaches supported the effects of NO in PR development and SCN maintenance at different levels (Fig. 1C). NO affects PR length as the loss of function of GSNOR, a central regulator of SNOs, has a striking impact on root architecture, causing significant reduction of root length (Kwon *et al.*, 2012). Additionally, the depletion of endogenous NO by the scavenger 2-(4-carboxy-phenyl)-4,4,5,5-tetramethylimidazoline-1-oxyl-3-oxide (cPTIO) results in a significant increase of PR length, while the NO donor sodium nitroprusside (SNP) decreased PR growth in a dose-dependent manner in tomato seedlings (Correa-Aragunde *et al.*, 2004). Although the different NO-overproducing mutants of *cue1* showed an altered meristem organization of the stem cells around the QC depending on the developmental stage of the plant (Fernández-Marcos *et al.*, 2011; Lechón *et al.*, 2020), treatments with exogenous NO donors produced a deregulation in the organization of RAM cells (Fernández-Marcos *et al.*, 2011).

These effects have been shown at the transcriptional and protein levels. Transcriptomic studies reveal that stem cell-related genes, such as *CLAVATA3/EMBRYO SURROUNDING REGION-RELATED* (*CLE*), are differentially expressed after 6 h of treatment with the NO donor *S*-nitrosocysteine (CySNO) (Shahid *et al.*, 2019). The PHYTOCHROME INTERACTING FACTOR3 (PIF3) plays an important role in the inhibitory effect of NO on *A. thaliana* seedling root growth, where the effect of NO appears at the protein level (Bai *et al.*, 2014). Hence, NO can be considered as a messenger molecule with a variety of effects on the regulation of gene expression and/or PTMs of proteins pertaining to the configuration of the PR architecture and SCN maintenance.

### Adventitious root development

ARs become relevant during nutrient uptake, plant propagation, and stress responses (Steffens and Rasmussen, 2016). These structures are formed during post-embryonic *de novo* development and emerge from organs such as hypocotyls or tissues such as the pericycle (Falasca and Altamura, 2003; Li *et al.*, 2009). In agreement with other root types discussed in this review, NO is also involved in the development of ARs (Fig. 1A). The effect of this gasotransmitter on AR formation has been studied mainly through treatments with exogenous NO donors (SNAP, SNP, or GSNO). NO increases the number and length of ARs in cucumber, *Panax ginseng*, *Vigna radiata*, and mung bean in a dose-dependent manner, as higher concentrations of this gasotransmitter can inhibit AR development. In contrast, in the presence of an NO scavenger (cPTIO), ARs are reduced in the aforementioned species (Pagnussat *et al.*, 2002; Lanteri *et al.*, 2008; Tewari *et al.*, 2008; Sharma *et al.*, 2019). While an SNP-only treatment in *A. thaliana* does not produce this effect on AR formation, combined SNP and cadmium treatments promote the increase of ARs, suggesting a promoting effect of cadmium on NO effects in these species (Della Rovere *et al.*, 2022). Regarding the interaction with phytohormones, NO mediates Aux response during the AR formation in cucumber, and this induction can be either NO-mediated cGMP-dependent or cGMP-independent (Pagnussat *et al.*, 2003; Tewari *et al.*, 2008). In addition, the mitogen-activated protein kinase (MAPK) cascade is activated during this process by the latter pathway mentioned above (Pagnussat *et al.*, 2004). BRs have recently been demonstrated to be involved in NO-mediated AR development (Y. Li *et al.*, 2020). In this regard, BRs regulate endogenous NO production by activating enzymes related to NO synthesis (NOS-like and NR) during this process in cucumber. This activation is due not only to increased enzymatic activity but also to the up-regulation of NR gene expression (Y. Li *et al.*, 2020). The promotion of AR formation because of exogenous BR treatment is also observed in *A. thaliana* (Della Rovere *et al.*, 2022). There are other molecules modifying the NO regulation of AR development, such as H_2_O_2_ and H_2_. Indeed, H_2_O_2_ acts downstream of NO signaling in mung bean and chrysanthemum AR formation (Li and Xue, 2010; Liao *et al.*, 2010). However, NO is known to act downstream of H_2_, being involved in cell cycle progression, up-regulating *CYCA*, *CYCB*, *CDKA*, and *CDKB* genes involved in this stage of AR development (Zhu *et al.*, 2016). In addition, there is H_2_-mediated induction of AR formation through regulation of *PM H*^*+*^*ATPase* and *14-3-3* gene expression and protein interaction (C. Li *et al.*, 2020). Finally, a molecular approach has identified the contribution of NO during AR development through *S*-nitrosylation of key proteins involved in cellular metabolism, transcription, translation, and signaling (Niu *et al.*, 2019).

### Lateral root development

LRs contribute to water and nutrient uptake, increasing the surface area and determining the radial size of the root system. LRs originate from the pericycle (Dolan *et al.*, 1993) and their number is not pre-determined, but depends on the integration of environmental cues (Malamy and Ryan, 2001). LR emergence is mainly controlled by Aux (Casimiro *et al.*, 2003) and tightly modulated by NO, which can integrate different aspects of Aux-mediated signaling pathways.

The involvement of Aux during LR formation has been described several years ago. Exogenous Aux application promotes LR initiation in *A. thaliana* (Reed *et al.*, 1998; Casimiro *et al.*, 2001) and tomato (Dubrovsky *et al.*, 2008), where its local accumulation in root pericycle cells lead to the acquisition of LR founder cell identity. In other plant species such as maize, these treatments have dual effects depending on the dose, and their modulation is linked to founder cell length (Alarcón *et al.*, 2019).

NO has a clear effect on LR initiation and growth, both under normal conditions and in response to abiotic stresses, but more information is needed on the specific molecular mechanisms involved in the regulation of this process. Using fluorescent probes, endogenous NO has been localized in LR primordia during all stages of development (Correa-Aragunde *et al.*, 2004; Kolbert *et al.*, 2008). Application of NO donors to tomato seedlings increased LR initiation and elongation in a dose-dependent manner, even in the presence of the Aux transport inhibitor *N*-1-naphthylphthalamic acid (NPA) (Correa-Aragunde *et al.*, 2004). Consistent with an NO-derived effect, treatments with the scavenger cPTIO were able to prevent LR induction by the synthetic Aux 1-naphthylacetic acid (NAA) (Correa-Aragunde *et al.*, 2004). In addition, indole-3-butyric acid (IBA) can induce NO production via NR (Kolbert *et al.*, 2008), highlighting a network between Aux and NO. Interestingly, the Aux-responsive PHB3, previously implicated in NO synthesis (Wang *et al.*, 2010), has been recently linked to LR primordium initiation (Li *et al.*, 2022; Luo *et al.*, 2022). It is worth mentioning that NO may have a dual effect depending on the portion of the root analyzed during the exogenous NO treatments (Lira-Ruan *et al.*, 2013). In response to abiotic stresses, the synergy between Aux and NO has also been described in rice, controlling LR formation to minimize toxic effects of cadmium and arsenic (Piacentini *et al.*, 2020), or under salt stress in sunflower (Singh and Bhatla, 2018). Other molecules that induce LR formation have been described in close association with NO, such as carbon monoxide (Guo *et al.*, 2008), alkamides (Méndez-Bravo *et al.*, 2010), carbon dioxide (Wang *et al.*, 2013), and nitrate (Sun *et al.*, 2018) (Fig. 1B).

All these results provide compelling evidence for the essential role of NO in coordination with Aux and reveal a complex network of components that shape and adjust the architecture of the root system during LR development.

### Nitric oxide signaling in plant regeneration

Plants have a great capacity to regenerate from small tissues or after damage or injury, re-establishing functional meristems by changing their developmental cell fate, restarting cell proliferation and differentiation (Birnbaum and Sánchez-Alvarado, 2008), and re-establishing functional organs (Ikeuchi *et al.*, 2019). In fact, regeneration often triggers the formation of a callus, a disorganized mass of growing undifferentiated cells capable of producing new tissues throughout cell differentiation (Bidabadi and Jain, 2020).

The transition from cell proliferation to differentiation is controlled by several molecular and hormonal mechanisms (Tsukagoshi *et al.*, 2010). The main regulation is exerted by hormonal gradients (Birnbaum and Sánchez-Alvarado, 2008) that mediate transcriptional responses (Efroni *et al.*, 2016). The combined induction of Aux and CKs, followed by their spatial separation, activates specific genetic programs that enable root SCN formation (Efroni *et al.*, 2016). Nevertheless, other hormones participating in the process are BRs (Ackerman-Lavert *et al.*, 2021; Takahashi and Umeda, 2022) that control cell proliferation as observed by cell cycle markers (González-García *et al.*, 2011); ethylene (ET) which promotes cell renewal after stem cell loss through a heterodimeric transcription factor complex formed by ETHYLENE RESPONSE FACTOR115 (ERF115), and others (Heyman *et al.*, 2016); ERF115 is also induced by jasmonic acid (JA). Additionally, JA promotes stem cell activation and regeneration through the RETINOBLASTOMA-RELATED (RBR)–SCARECROW (SCR) network and ERF115 (Zhou *et al.*, 2019).

Other signaling stimuli are involved in plant regeneration, such as oxidative stress (Libik *et al.*, 2005; Vijendra *et al.*, 2017) and ROS (Ikeuchi *et al.*, 2018) that control cell differentiation and proliferation (Tsukagoshi *et al.*, 2010), but their roles in plant regeneration are largely unknown (Ikeuchi *et al.*, 2018). In *A. thaliana*, the thioredoxin DCC1 determines the shoot regeneration capacity. Loss of DCC1 function triggers ROS production, inhibiting regeneration compared with wild-type plants where low ROS levels promote the process (Zhang *et al.*, 2018) (Fig. 1D). Other studies have revealed that phospholipid hydroperoxide glutathione peroxidase (PHGPx) regulates shoot regeneration in *Nicotiana tabacum* (Faltin *et al.*, 2010) (Fig. 1D). These findings showed that ROS are involved in the regulation of *de novo* organogenesis. Indeed, superoxide regulates stem cell fate through the homeodomain transcription factor WUSCHEL (WUS), expressed in shoot apical meristem (SAM) niche cells (Zeng *et al.*, 2017). Still, the role of other RNS signaling molecules, such as NO, in plant regeneration and their interplay with other hormones in *de novo* organogenesis is largely unknown. In animal cells, NO has been reported to modulate stem cell survival or differentiation (Midgley *et al.*, 2020), supporting results obtained with the moss *Physcomitrella patens* where NO regulates protonema regeneration from the moss protoplasts (Cervantes-Pérez *et al.*, 2020) (Fig. 1E). In fact, exogenous treatments with NO donors decrease the number of cells per protonema and inhibit the cell cycle (Cervantes-Pérez *et al.*, 2020). Furthermore, NO mediators have been suggested in the regulation of SCN regeneration after the surgical removal of the QC, via PGBs in *Zea mays* (Mira *et al.*, 2020) (Fig. 1F). Despite recent findings on the role of NO in plant SCN homeostasis and cell differentiation (Sanz *et al.*, 2014), the mode of action of NO in the re-specification of functional SCNs during regeneration remains to be elucidated.

## Interaction of phytohormones and nitric oxide in the regulation of root system architecture

NO is known to interact with plant hormones and other endogenous molecules, and can affect their biosynthesis, catabolism, transport, perception, and signal transduction (reviewed in Sanz *et al.*, 2015). In the following sections we explore the physiological and molecular insights into the control of root development and regeneration through the interaction between NO and Auxs, CKs, BRs, or SLs.

### Auxins

**Fig. 2.**
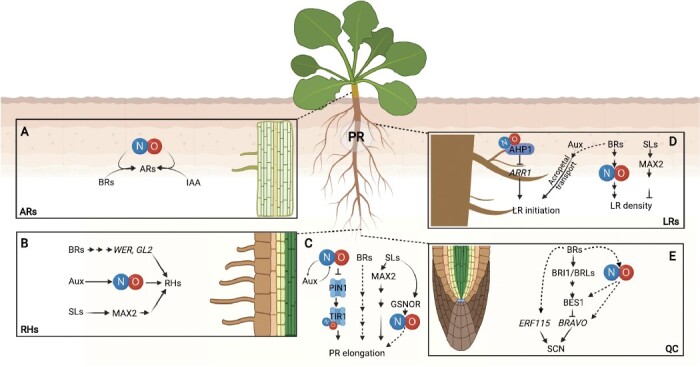
Phytohormons and NO interaction during the regulation of the root system architecture. (A) NO, in cooperation with Aux and BR signaling components, regulates AR development by still unknown mechanisms. (B) NO acts downstream of Aux during root hair (RH) formation. NO-overproducing mutants or the addition of exogenous NO increase Aux levels. BRs and SLs also contribute to RH formation and growth, respectively. (C) In PR development, NO regulates PR elongation through direct crosstalk with Aux at different levels. NO inhibits rootward auxin transport by negatively regulating accumulation of the Aux transporter PIN1, and the *S*-nitrosylated Aux receptor TIR1 highly interacts with auxin/IAA proteins. SLs can regulate the abundance and activity of GSNOR and modify the endogenous NO levels that affect PR elongation. (D) NO negatively regulates CK signaling by inhibiting the phosphorelay activity through *S*-nitrosylation of AHP1 at Cys115, repressing its phosphorylation and subsequent transfer of the phosphoryl group to ARR1. BR treatment induces the endogenous NO production and increases LR density, a process also regulated by SLs in an opposite manner. (E) BRs control SCN activity in the root meristem via BES1 inhibition of *BRAVO* expression. Jointly, BRs also positively control the transcription factor ERF115 for a proper SCN activity in the root. NO might have an impact on the BR signaling pathway to regulate meristem activity (created with BioRender.com).

Auxs are essential for almost every aspect of plant growth and development, and roots are no exception. The first reports of Aux action on root formation date back to the 1930s with the work of Thimann and Went (Overvoorde *et al.*, 2010) and, since then, multiple studies have assessed this regulation. To date, some of the best characterized Aux-associated phenotypes include the dose-dependent increase in the length of epidermal-derived root hairs and LR primordia, the effect of Aux concentration on PR length, and the response to gravity (Pitts *et al.*, 1998; Rahman *et al.* 2002; Ishida *et al.* 2008; Péret *et al.* 2009; Overvoorde *et al.*, 2010) (Fig. 2).

As mentioned in previous sections, NO is involved in the regulation of root formation and architecture, a phenomenon first observed by Gouvêa *et al.* (1997), who found that NO induces cell elongation in a similar way to Aux. Since then, multiple studies continue to evaluate the relationship of NO with Aux in root development and architecture. In Pagnussat *et al.* (2002), the authors suggested that NO could mediate the Aux response in IAA-induced AR development (Fig. 2A). To date, there is plenty of evidence that NO acts downstream of Aux during the formation of root hairs and LRs (Lombardo *et al.*, 2006; Wang *et al.*, 2010; Cao *et al.*, 2017) but, conversely, there are also reports indicating that NO-overproducing mutants or the addition of exogenous NO increase Aux levels depending on the tissue analyzed (Sánchez-Vicente *et al.*, 2021) (Fig. 2B).

NO inhibits rootward Aux transport in *A. thaliana* by reducing the amount of PIN-FORMED1 (PIN1) through a proteasome-independent post-transcriptional mechanism (Fernández-Marcos *et al.*, 2011). Other studies have demonstrated that the Aux receptor TRANSPORT INHIBITOR RESPONSE1 (TIR1) undergoes *S*-nitrosylation at two particular Cys residues, and this *S*-nitrosylation of TIR1 seems to promote its interaction with Aux/IAA proteins (Terrile *et al.*, 2012) (Fig. 2C). The NO donor GSNO has also been described to affect the polar distribution of PIN2 during gravitropism (Ni *et al.*, 2017; Paris *et al.*, 2018).

Roots of the NO-deficient mutant *nia1nia2noa1* display small meristems with abnormal divisions. Analysis of expression of the Aux synthetic reporter *DR5* in the NO-deficient mutants *noa1*, *nia1nia2*, and *nia1nia2noa1* showed reduced expression in all genotypes, indicating that Aux response is diminished in NO-deficient mutants (Sanz *et al.*, 2014). Furthermore, the inhibitory effects of Aux on root growth were partially suppressed in NO-deficient plants. On the other hand, PGB mutants, which have higher endogenous NO levels, have impaired Aux metabolism, resulting in drastic alterations in embryogenesis and root development (Elhiti *et al.*, 2013).

More recently, it has been described in *A. thaliana* that external Aux stimuli can induce the root clock that regulates lateral organ spacing along the PR through oscillating gene expression (Perianez-Rodriguez *et al.*, 2021). Whether NO contributes to this process in some way due to its close relationship with Aux could be an area of study worth exploring.

### Cytokinins

CKs are adenine derivatives identified in 1957 by Skoog and Miller as compounds that act antagonistically to Aux in tobacco cell cultures (Bishopp *et al.*, 2011). CKs also play an important role in the development and architecture of the root system. Studies have examined their function and interaction with Aux in several ways. CKs and Aux also have antagonistic effects on root development. CKs interfere with LR development and patterning by influencing Aux transport and homeostasis (reviewed in Orman-Ligeza *et al.*, 2013). Alterations in the production, perception, or signal transduction of CKs affect the organogenesis of LRs (Laplaze *et al.*, 2007; Bielach *et al.*, 2012b). CKs promote PIN1 depletion in specific polar domains and consequently reorganize cellular PIN polarities and directly regulate the direction of Aux flux (Marhavý *et al.*, 2014).

Overexpression of the enzyme CYTOKININ OXIDASE (CKX) led to degradation of CK and reduced levels of CKs, resulting in increased root branching (Werner *et al.*, 2001, 2003). Furthermore, *A. thaliana CKX* genes are expressed in LR primordia, suggesting that removal of the CK signal is important for the progression of LR development (Schmülling, 2002; Werner *et al.*, 2003). In contrast, *A. thaliana* mutants defective in CK receptors or response regulators (ARRs) required for CK response displayed increased root branching (To *et al.*, 2004).

Through a genome-wide association study, it was revealed that signal peptide processing of CYTOKININ OXIDASE 2 (CKX2) affects its enzymatic activity and, consequently, the degradation of CKs in *A. thaliana* (Waidmann *et al.*, 2019). CK signaling interferes with growth at the upper flank of LRs and thus prevents downward bending. It has been proposed that Aux and CK signaling occur in opposing organs and, as a result, the flanks counteract each other’s negative effects on growth, suppressing organ growth towards gravity and allowing radial expansion of the root system. An analysis of CK levels in roots suggests that spatiotemporally regulated metabolic processes contribute to a differential distribution of CK derivatives along the PR. Specifically, LR initiation and primordium formation take place in root zones with high levels of active CKs; however, LR emergence occurs in root zones where biologically inactive CKs accumulate (Bielach *et al.*, 2012a, b).

Considering the importance of CKs in root architecture and their close interaction with Aux, it would not be surprising if NO, directly or through Aux function, is also influencing the function of CKs in root architecture. For example, Liu *et al.* (2013) observed how the double mutants of *continuous NO* (*cnu1-2*) and *nitric oxide overexpression 1* (*nox1*) have reduced severity of NO level phenotypes. A similar effect was observed when treating the *nox1* mutant with *trans*-zeatin. Moreover, it was also observed that peroxynitrite can react with zeatin *in vitro*, suggesting that CKs suppress the action of NO through direct interaction with each other (Liu *et al.*, 2013). Similarly, NO negatively regulates CK signaling by inhibiting the phosphorelay activity through *S*-nitrosylation. *S*-Nitrosylation of AHP1 at Cys115 represses its phosphorylation and subsequent transfer of the phosphoryl group to ARR1 (Feng *et al.*, 2013) (Fig. 2D).

Although the importance of Aux and CKs has been known for decades, suggesting a complex network of signaling interactions, the molecular mechanisms underlying their effects is a field with great potential and relevance, even more so if we consider the role of NO and its effect in many regulatory networks in plants.

### Brassinosteroids

Plants and animals share a group of chemically related types of very important signaling molecules, the steroid hormones (Picard *et al.*, 1990; Clouse, 2011). Plant BRs, and the most active version brassinolide, were identified as plant growth-promoting hormones isolated from *Brassica napus* pollen (Grove *et al.*, 1979). BRs control cell expansion, division, and differentiation (Clouse, 2011), regulating many different stages of plant growth (Albertos *et al.*, 2022a) and stress responses (Albertos *et al.*, 2022b). Genetic and chemical evidence has linked BRs to root growth through control of meristem size. In *A. thaliana*, BRs promote root elongation under very low concentrations but inhibit root growth at high concentrations (Müssig *et al.*, 2003; González-García *et al.*, 2011). Well-balanced BR homeostasis and signaling is necessary for the control of cell cycle progression and differentiation in the root meristem for optimal root growth and development in a cell-specific manner (González-García *et al.*, 2011; Fàbregas *et al.*, 2013). The presence of BR receptors BRI1 (BRASSINOSTEROIDS INSENSITIVE 1), expressed almost ubiquitously in the root, and its homologs BRI1-LIKE 1, 2, and 3 within vascular stem cells indicates the relevance and variety of BR signaling pathways in the architecture of root morphology. The function of BRs within the root SCN ultimately depends on the modulation of the transcription factor BRAVO (BRASSINOSTEROIDS AT VASCULAR AND ORGANIZING CENTER) (Vilarrasa-Blasi *et al.*, 2014). BRs balance *BRAVO* expression through its negative regulation by the BR-regulated transcription factor BES1, repressing cell divisions in the QC (Vilarrasa-Blasi *et al.*, 2014). In addition, the transcription factor ERF115, associated with the regulation of cell divisions and maintenance of the SCN after cell damage, is positively regulated by BRs (Heyman *et al.*, 2013). Interestingly, BR signaling levels increase proportionally closer to the differentiation/elongation zone, and meristem size may be sufficiently controlled only by BR perception in the epidermis (Hacham *et al.*, 2011). Moreover, BRs have been described as important regulators of root hair formation. BRs control the expression of *WEREWOLF* (*WER*) and *GLABRA2* (*GL2*), essential for position-dependent epidermal cell fate specification in roots (Kuppusamy *et al.*, 2009). It should be emphasized that the regulation of LR development combines the modes of action of BRs and Aux. A concentration-dependent response of BRs can promote, at low doses, or suppress, at high doses, LR initiation by modifying acropetal Aux transport (Bao *et al.*, 2004; Gupta *et al.*, 2015). In all BR-regulated processes that define the architecture of the root system, the maintenance of the RAM and the development of ARs and LRs, the signaling molecule NO has also been described as an important modulator (Fig. 2) (Fernández-Marcos *et al.*, 2011, 2012; Sanz *et al.*, 2014). For example, exogenous treatments with BRs and NO, and the combination of both molecules, promoted adventitious rooting in cucumber plants (Y. Li *et al.*, 2020) (Fig. 2A). In *A. thaliana*, BR treatments increased LR density, and this correlated with NO function (Tossi *et al.*, 2013) (Fig. 2D). In these studies, the effect of BRs on root architecture was subjected to the induction of endogenous NO production promoted after BR treatment. However, the direct role of NO in the different molecular players controlled by BRs in root signaling remains to be elucidated (Fig. 2E). It will be of great relevance to further study which of the mentioned transcription factors might be tightly controlled by the interaction between BR signaling and NO-dependent post-translational modifications such as *S*-nitrosylation.

### Strigolactones

A new class of plant hormones, first identified as shoot branching factors, are the plant terpenoids SLs (Gomez-Roldan *et al.*, 2008; Umehara *et al.*, 2008). For >50 years, some terpenoid compounds used as germination stimulants in parasitic plants were finally identified as SLs (Cook *et al.*, 1966; Xie *et al.*, 2010). In addition to shoot branching, SLs can control plant growth and development and stress responses (Pandey *et al.*, 2016). In terms of root architecture, SLs regulate PR and root hair elongation and LR density (Kapulnik *et al.*, 2011; Koltai, 2011) (Fig. 2B–D). Furthermore, SLs have been suggested to mediate root responses to the environment due to their role in symbiotic interactions and nutrient stresses (Akiyama *et al.*, 2005; López-Ráez *et al.*, 2008; Ruyter-Spira *et al.*, 2011). Exogenous applications of SLs promote PR elongation in a dose response- and nutrient content-dependent manner through the specific cooperation of MAX2 (MORE AXILLARY BRANCHES 2), a SKP1-CULLIN-F-BOX (SCF) complex, one of the main components of the SL signaling cascade (Jain *et al.*, 2007; Ruyter-Spira *et al.*, 2011). An opposite function in the regulation of LR formation has been suggested, where MAX2 may be also involved as a key player. Deficient or signaling SL mutants have increased LR density, and exogenous SL treatments reduce LR formation (Kapulnik *et al.*, 2011; Ruyter-Spira *et al.*, 2011). As for root hairs, SLs lead to increased root hair length, a process again mediated through the master regulator MAX2 (Kapulnik *et al.*, 2011). However, the effect of SLs on root architecture has been found to depend on the interaction with other hormones and plant growth regulators (Beveridge and Kyozuka, 2010; Dun *et al.*, 2012). Although the current knowledge is still limited, new discoveries have shown an interaction between SLs and the gasotransmitter NO in the formation of a suitable root architecture (Fig. 2). Using chemical and genetic approaches, it was possible to elucidate the role of SLs in the regulation of the abundance and activity of the enzyme GSNOR, which is responsible for the production of part of the endogenous SNOs in *A. thaliana* plants (Fig. 2C) (Oláh *et al.*, 2020, 2021). It was suggested that functional GSNOR activity is required to control NO levels during SL-induced PR elongation. Alternatively, a joint mechanism between NO and SLs in rice was proposed to regulate root elongation under nutrient deficiency (Sun *et al.*, 2016). Future studies are needed to identify the interaction between SL and NO signaling for a fine-tuned regulation of root architecture.

## Nitric oxide dynamics during root hypoxia

Hypoxia, one of the most important abiotic stresses, is primarily caused by flooding. Usually, during flooding events only the root system is submerged (i.e. waterlogging), so the effects of hypoxia and anoxia on roots are better understood than those on shoots. Hypoxia-related changes in overall root anatomy and physiology vary between plant species and can include the formation of aerenchyma (Drew *et al.*, 2000) or ARs as escape mechanisms; and growth cessation as a quiescence mechanism (reviewed in Voesenek and Bailey-Serres, 2015).

**Fig. 3.**
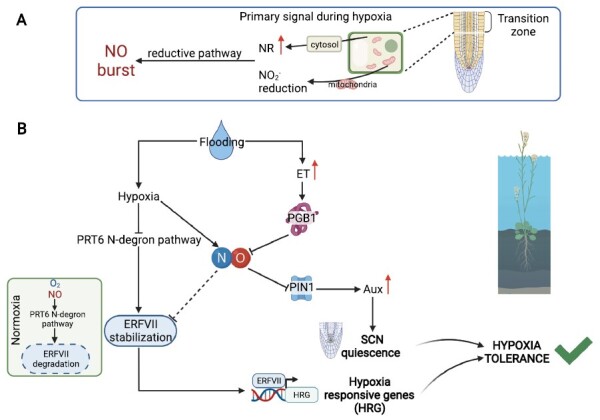
NO dynamics during hypoxic stress in roots. (A) NO biosynthesis during hypoxia. An NO burst occurs in the roots, specifically in the transition zone of the PR. The increase of NO comes mainly from the reductive pathway. Nitrite (NO_2_^–^) is reduced in the cytosol by the enzyme NR and in the mitochondrial matrix, where NO_2_^–^ acts as an alternative electron acceptor. (B) NO regulation and signaling during hypoxia. With the establishment of hypoxia (i.e. flooding), the phytohormone ET accumulates within the root. ET induces the expression of the NO-scavenger PGB1. The reduction of NO levels promotes an Aux accumulation through changes in PIN1 localization, which ultimately leads to a retention of a quiescent state in the SCN. The reduction of NO levels, along with hypoxia, promotes ERFVII group stabilization and the induction of the expression of hypoxia-responsive genes. NR, nitrate reductase; PGB1, phytoglobin1; ET, ethylene; Aux, auxin (created with BioRender.com).

With the establishment of the hypoxic stress, a burst of NO occurs in the roots. NO biosynthesis by nitrite reduction at the mitochondrial membrane is the main source of NO production in roots under hypoxia or anoxia (Planchet *et al.*, 2005; Gupta and Igamberdiev, 2011). However, the contribution of the oxidative pathway to NO biosynthesis is reduced by the lack of oxygen during hypoxic stress. The enzyme NR catalyzes in the cytosol the reduction of nitrate to nitrite and then to NO in an NAD(P)H-dependent reaction. As both NR activity and expression increase during hypoxia (Botrel and Kaiser, 1997; Klok *et al.*, 2002; Morard *et al.*, 2004), nitrite accumulation leads to its translocation from the cytosol to the mitochondria where it is reduced to NO at the complex III, cytochrome *c* oxidase (COX), and alternative oxidase (AOX). The use of nitrite as an alternative electron acceptor also contributes to limited ATP synthesis under nitrite supply (Stoimenova *et al.* 2007; Gupta *et al.*, 2020) (Fig. 3A).

To prevent an overaccumulation of NO, a scavenging system exists, in which the hemoprotein PGB plays a major role. PGBs have a high affinity for NO (Duff *et al.*, 1997), and their expression increases rapidly during hypoxic stress (Dordas *et al.*, 2003, 2004; Gasch *et al.*, 2016). PGB1 uses NO to form nitrate in the so-called PGB–NO cycle (Perazzolli *et al.*, 2004; Igamberdiev *et al.*, 2005). This cycle, in addition to curbing high NO levels, also prevents an over-reduction of the NAD^+^ and generates low levels of ATP.

Although the exact dynamics of NO in flooded plants remains to be elucidated, it is clear that the increase in NO during hypoxia is one of the primary signals emitted by plants during this stress and undeniably has functional implications for plant survival (Mugnai *et al.*, 2012; Gupta and Igamberdiev, 2016; Mira *et al.*, 2016). NO can also exert a direct regulatory role, through post-translational modification of proteins (*S*- or metal-nitrosylation and Tyr nitration). Some of the targets of NO are COX, aconitase, PGB, and several antioxidant enzymes (Perazzolli *et al.*, 2004; Gupta *et al.*, 2012; Romero-Puertas *et al.*, 2013; Holzmeister *et al.*, 2015; Yang *et al.*, 2015; Begara-Morales *et al.*, 2016; Jain and Bhatla, 2018).

Research on root meristem alterations during hypoxia is limited due to the difficulties in visualizing and analyzing responses. Previous works revealed that maize root tips exposed to 4% oxygen for 24 h (Mira *et al.*, 2016) or *A. thaliana* root tips exposed to 1% oxygen for 4 h (Hartman *et al.*, 2019) undergo cell death.

In maize, overexpression of *ZmPgb1.1* or *ZmPgb1.2* confers increased resistance to hypoxia or waterlogging stress by influencing the redox state of the QC. Overexpression of *ZmPbg1.1* reduces NO levels, which influences PIN1 localization and thus Aux distribution, preserving an oxidized environment in the QC (Mira *et al.*, 2020). Regarding root regeneration, lines overexpressing *ZmPgb1.1* have more capacity to regenerate a new QC in hypoxic roots after QC excision than the wild-type roots. Lines overexpressing *ZmPgb1.2* also confer more resistance to waterlogging stress. Transcriptomic studies of these lines using RNAseq revealed that this overexpression up-regulates genes involved in JA and ET signaling, abscisic acid (ABA) metabolism, and activation of the fermentation pathway (Youssef *et al.*, 2019). Also, in maize, local NO production in the transition zone (TZ), which is located between the RAM and the basal elongation region, is indispensable for the systemic induction of fermentation activity that confers acclimation to hypoxia throughout the root (Mugnai *et al.*, 2012).

In *A. thaliana*, root tip survival experiments have been conducted to evaluate RAM survival during hypoxic stress (Hartman *et al.*, 2019; Liu *et al.*, 2022). While root tips died after 3–4 h of severe hypoxia, ET or submergence (which induces ET accumulation) pre-treatments increased RAM survival. ET induces *PGB1* expression, which scavenges NO, favoring ERFVII stabilization through inactivation of the PRT6 N-degron proteolytic pathway (Gibbs *et al.*, 2014; Hartman *et al.*, 2019). The stable ERFVII group migrates to the nucleus, where it activates the transcription of hypoxia-responsive genes (Licausi *et al.*, 2011; Gibbs *et al.*, 2014) (Fig. 3B).

In summary, during hypoxia, the NO levels are finely modulated by production and removal mechanisms that guarantee early detection of low oxygen levels and adequate activation of hypoxia response.

## Concluding remarks

Despite the new advances achieved in knowledge of NO regulation of post-embryonic root organogenesis, future studies are required to understand how NO controls the complex molecular mechanisms of root development and regeneration and its crosstalk with hormones in plants.

Thus, NO and ET gasotransmitters are considered multifunctional plant signals linked at the synthesis level but antagonistic in the regulation of plant growth and development (Kolbert *et al.*, 2019). Recent evidence supports the relevant role of ET during soil compaction (Pandey *et al.*, 2021) and hypoxia caused by flooding events where the root system is submerged (Hartman *et al.*, 2019; Liu *et al.*, 2022), due to reduced gas diffusion. Deciphering the mechanism of NO sensing, through direct binding of the molecule, and the post-translational regulation of molecular targets of these interconnected signaling pathways will shed light on the control of root development and regeneration, which will be of key relevance for plant productivity.

A landmark aspect for future research is the identification of the elements and molecular bases involved in root development and regeneration caused by hypoxic stress responses, essential for understanding their function in the plant, which is a prerequisite for their genetic improvement.
